# A primary health care model for managing pre-eclampsia and eclampsia in low- and middle- income countries

**DOI:** 10.1186/s12978-020-0897-0

**Published:** 2020-04-06

**Authors:** Charlotte E. Warren, Sharif Mohammed Ismail Hossain, Salisu Ishaku, Deborah Armbruster, Emily Hillman

**Affiliations:** 1 Population Council, Washington, DC, USA; 2 Population Council, Dhaka, Bangladesh; 3 Julius Centre for Health Sciences and Primary Care, UMC, Utrecht, The Netherlands; 4 USAID, Washington DC, USA

## Abstract

**Background:**

Hypertensive disorders in pregnancy, specifically pre-eclampsia and eclampsia (PE/E), are the second biggest killer of pregnant women globally and remains the least understood and most challenging maternal morbidity to manage. Although great strides were made in reducing maternal and newborn mortality between 1990 and 2015, this was clearly not enough to achieve the global health goals. To reduce maternal deaths: 1) early detection of PE needs to be improved; 2) effective management of PE/E needs to occur at lower health system levels and should encourage timely care-seeking; and 3) prioritizing the scale up of a comprehensive package of services near to where women live.

**Findings:**

This commentary describes a pragmatic approach to test scalable and sustainable strategies for expanding access to quality under-utilized maternal health commodities, interventions and services. We present a primary health care (PHC) PE/E Model based on implementation research on identified gaps in care in several countries, accepted global best practice and built on the basic premise that PHC providers can take on additional skills with adequate capacity building, coaching and supervision, and community members desire control over their own health. The PHC PE/E model displays the linkages and opportunities to prevent and treat PE/E in a simplified way; however, there are numerous interlinking factors, angles, and critical points to consider including leadership, policies and protocols; relevant medicines and commodities, ongoing capacity building strategies at lower levels and understanding what women and their communities want for safe pregnancies.

**Conclusion:**

The PHC model described here uses PE/E as an entry to improve the quality of ANC and by extension the pregnancy continuum. Bringing preventive and treatment services nearer to where pregnant women live makes sense.

## Abstract (fr)

**Contexte:**

Les troubles hypertensifs pendant la grossesse, en particulier la pré-éclampsie et l’éclampsie (PE/E), constituent la deuxième cause de mortalité maternelle dans le monde et est. la moins bien comprise et la plus difficile à gérer. Bien que de grands progrès aient été réalisés dans la réduction de la mortalité maternelle et néonatale entre 1990 et 2015, il est. clair que cela n’a pas suffi pour atteindre les objectifs mondiaux en matière de santé. Pour réduire la mortalité maternelle: 1) la détection précoce de la PE doit être améliorée, 2) la gestion efficace de la PE/E doit se faire à des niveaux inférieurs du système de santé et devrait encourager la recherche de soins en temps utile et 3) la priorité doit être donnée à l’élargissement d’un ensemble complet de services à proximité du lieu de résidence des femmes.

**Résultats:**

Ce commentaire décrit une approche pragmatique visant à tester des stratégies évolutives et durables pour élargir l’accès à des produits, interventions et services de santé maternelle de qualité et sous-utilisés. Nous présentons un modèle PE/E de soins de santé primaires (SSP) basé sur la recherche de mise en œuvre des lacunes identifiées dans les soins dans plusieurs pays, sur les meilleures pratiques mondiales acceptées et sur le principe de base selon lequel les prestataires de SSP peuvent acquérir des compétences supplémentaires avec un renforcement des capacités, un encadrement et une supervision adéquats, et les membres de la communauté souhaitent contrôler leur propre santé. Le modèle PE/E de SSP présente les liens et les possibilités de prévenir et de traiter la PE/E de manière simplifiée. Cependant, il existe de nombreux facteurs, angles et points critiques liés entre eux à prendre en compte, notamment le leadership, les politiques et les protocoles, les médicaments et les produits pertinents, les stratégies de renforcement des capacités en cours aux niveaux inférieurs et la compréhension de ce que les femmes et leurs communautés souhaitent pour des grossesses sans risque.

**Conclusion:**

Le modèle de SSP décrit ici utilise la PE comme entrée pour améliorer la qualité des soins prénataux et par extension le continuum de la grossesse. Il est. logique de rapprocher les services de prévention et de traitement du lieu de résidence des femmes enceintes.

## Key message

This model provides a pragmatic approach for improving access to antenatal services for detection, prevention and management of pre-eclampsia and eclampsia at the primary health care level.

## Background

In October 2018, in Astana, Kazakhstan, the global health community reaffirmed their commitment towards universal health coverage and made primary health care (PHC) a cornerstone of their attempt to reach the sustainable development goals (SDGs) by 2030 [1]. Although great strides were made in reducing maternal and newborn mortality between 1990 and 2015 (by 44 and 49% respectively), this was not enough to achieve the SDGs [2, 3]. More than 830 women and 7700 newborns still die each day from pregnancy-related complications; an additional 7300 women experience a stillbirth with approximately 16% occurring in pregnancies complicated by hypertension [4]. Of the three direct causes of obstetric mortality (bleeding, sepsis and hypertensive disorders of pregnancy (HDPs) accounting for 27, 11 and 14% respectively) [5], HDPs, specifically pre-eclampsia and eclampsia (PE/E), remain the least understood and most challenging to manage [6]. Risks to mothers’ cardiovascular and cerebrovascular health decades later and the inter-generational impact of HDPs are only now starting to be understood and remain unquantified in low- and-middle-income countries [6, 7].

Building on the work and successful efforts to prevent and manage postpartum hemorrhage (PPH) [8, 9] over the last 10 years – donors and maternal health experts started to focus on other causes of maternal deaths. Globally pre-eclampsia (PE) is the second major killer of pregnant and postnatal women, but unlike PPH, where approximately 70% can be prevented by administering oxytocin, PE is harder to recognize early and the treatment, magnesium sulphate (MgSO_4_), is only given to those with severe disease. To reduce maternal and perinatal deaths: 1) early detection of PE needs to be improved; 2) effective management of PE/E needs to occur at lower health system levels and should encourage timely care-seeking; and 3) prioritizing the scale up of a comprehensive package of services near to where women live [10–14].

This commentary describes a pragmatic approach undertaken by a multi-country study (supported by USAID from 2014 to 2019 and implemented by Population Council, through the Ending Eclampsia project) to test scalable and sustainable strategies for expanding access to quality under-utilized maternal health commodities, interventions and services. The project brings an implementation science approach to catalyze focus on PE/E by building on evidence-based recommendations [15, 16]. To understand the evolution of the development of the PHC model for PE/E presented here, we need to understand the prevailing practices prior to 2014.

Findings from the multi-country ‘MAGPIE trial’ in 2001 demonstrated that MgSO_4_ reduces the risk of eclamptic seizures by half among women with pre-eclampsia and reduces death from eclampsia [15]. Based on these findings, countries registered MgSO_4_ on their Essential Medicines List (EML) and introduced it to referral facilities. Although WHO recommends MgSO_4_ as the anticonvulsant for managing severe PE/E, (and methyldopa and hydralazine for controlling severe hypertension in pregnancy) [16, 17], the burden of calculating dosage of MgSO4 is daunting, time-consuming and can introduce errors, tempting providers to substitute diazepam for MgSO4, a less effective anticonvulsant [8]. Moreover, MgSO_4_ toxicity may result in respiratory failure and the need for the antidote, calcium gluconate. Even though less than 0.1% women die due to toxicity, there is a “fear” among providers making them reluctant to use MgSO_4_ [18]. More recently global advocacy efforts through the UN Commission on Life Saving Medicines for Women and Children 2012–14 focused on procuring quality MgSO_4_ and only one formulation of MgSO_4_ (50% or 5 g in 10 ml) to reduce the calculation challenges that exist [8].

Following the more widespread uptake of MgSO_4_ in referral facilities, a few studies also demonstrated the feasibility and acceptability of task-sharing management of PE to lower levels where providers administer a loading dose of MgSO_4_ correctly and refer the patient for further management [6, 19, 20]. However, WHO recommendations on task shifting or sharing maternal and newborn health (MNH) services do not include use of antihypertensives for severe hypertension for lower level cadres to prescribe [21]. Many countries are guided by WHO and therefore do not allow these cadres to prescribe and administer life-saving drugs for pregnant women with obstetric complications, yet this is where women often first seek care. Although 29/31 countries surveyed had MgSO_4_ on their EML in 2011, there is limited information available on specific antihypertensives for use during pregnancy in these countries [17].

### Landscape analysis in five countries – baseline findings

In 2015–2016, we conducted landscape analyses in Bangladesh [10], Nigeria [11], and Pakistan [12] on access to PE/E services. Further in-depth assessments in Ethiopia and Kenya provided a broader picture of the provision and experience of care for PE/E across sub Saharan Africa and south east Asia. Key findings included providers’ poor knowledge and skills in addition to a lack of essential commodities and equipment. Facilities were more likely to have oxytocin for prevention and management of PPH than MgSO_4_ to manage PE/E. Specific gaps at the lower facility levels include lack of task shifting policy, weak referral policies, lack of access and use of antihypertensives; lack of postpartum monitoring; and lack of community awareness around PE/E including danger signs. Headaches and blurred vision associated with PE/E are perceived as malaria or ‘overthinking’ and swollen ankles are perceived as normal. Women who experienced PE/E describe multiple contacts with the health system before receiving the necessary life-saving care [10–12]. The learning from the country level landscape analyses, in collaboration with input from key stakeholders – including in-country Ministry of Health officials, partners working in MNH and discussions with members of national and international technical working groups – formed the basis of a comprehensive PHC model for PE/E, tested through implementation research between 2016 and 2018. The model also built on evidence-based recommendations from WHO and other international experts [15, 20, 22].

## The primary health care - pre-eclampsia/eclampsia model

**Fig. 1:**
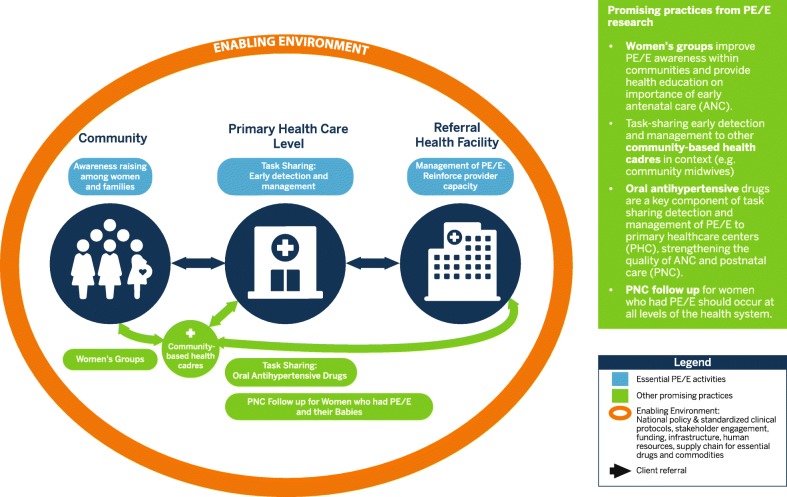
Primary Health Care - Preeclampsia /Eclampsia Model

We present a PHC PE/E Model (see Fig. 1) based on implementation research on identified gaps in care in several countries, accepted global best practice and built on the basic premise that PHC providers can take on additional skills with adequate capacity building, coaching and supervision, and community members desire control over their own health [1]. By using PE as a lens, we believe this model is transferrable across countries and practices along the pregnancy continuum with a particular focus on antenatal care (ANC). However, our research has also shown that without appropriate policies, sufficient finances, supplies/medicines in place and effective governance/leadership, the success of the model may be limited [10–12]. Here, we describe the PHC model for PE/E care representing a set of critical interventions for early detection and quality PE/E prevention and management tested through IR:

### Community level

- Use women’s groups as a platform to increase awareness on danger signs and importance of early care seeking during pregnancy (Cross River state, Nigeria).
- Include country-specific community-based cadres: Community Midwives (CMWs) in Sindh Province, Pakistan, identified as capable providers of MgSO_4_ loading dose with referral.

### Primary health care

- Use PE/E as a lens on quality of ANC:
  - ◦ Encourage early care seeking behavior for enabling early detection of high blood pressure (BP).
  - ◦ Raise awareness of signs and symptoms and what women should expect/ask at facilities during ANC and postnatal (PNC) contacts.
- Build capacity / improve performance of service providers:
  - ◦ Mobilize master trainers who are available locally.
  - ◦ Develop training plans, which include follow-up mentoring, coaching and supportive supervision, including refresher training as necessary during facility visits.
  - ◦ Ensure competent and confident providers can identify, monitor, stabilize and refer patients to higher levels.
- Ensure functionality and/or readiness of system:
  - ◦ Availability and use of equipment, and supplies for detection and treatment: BP machines, stethoscope, urine dipsticks, patella hammer, syringes, needles, etc.
  - ◦ Ensure consistent supply of quality medicines for managing HDPs; antihypertensives (those readily available in country – labetalol, nifedipine, or alpha methyldopa), MgSO_4_, and calcium gluconate.
  - ◦ Engage pharmacy workers and logistics officers to ensure they understand the importance of consistent stock of equipment and commodities for obstetric complications.
- Provide quality care:
  - ◦ Inform women and families routinely on danger signs/symptoms of pregnancy. This could be done through individual visits or through group counseling.
  - ◦ Check BP and urine albumin routinely to detect PE early [15, 22]
  - ◦ If high BP or PE is identified, monitor closely, provide antihypertensives as appropriate.
  - ◦ If BP is not controlled or severe PE/E ensues, stabilize with antihypertensive, loading dose of MgSO_4_ and refer to appropriate facility [16, 22, 23].
- Ensure hypertension is controlled:
  - ◦ Use of a simple algorithm/clinical protocol: monitor BP closely.
  - ◦ Prescribe anti-hypertensive for moderate to severe hypertension [24].
  - Ensure referral system is in place:
  - ◦ Assist or identify locally available transport.
  - ◦ Referral notification with higher level facility.
  - ◦ Ensure post-referral monitoring/follow-up.

### Referral health facility

- Functional referral facility (Emergency Obstetric and Neonatal Care):
  - ◦ Ensure maternity units have received training on identification and management of PE/E.
  - ◦ Ensure use of nationally/internationally recognized treatment algorithms/protocols.
  - ◦ Ensure necessary equipment, and commodities, in place.
  - ◦ Ensure expertise and appropriate skills mix of staff available.
- Skilled providers to manage PE/E:
  - ◦ Ensure providers are confident and capable to manage PE/E appropriately (as above).
  - ◦ Assist local staff to create a functioning referral and counter referral plan.
- Monitor women with PE/E closely post-delivery and postpartum:
  - ◦ Ensure they are not discharged during the first 48 h or until BP is stabilized.
  - ◦ Ensure monitoring and postnatal follow-up care of women who experienced PE/E.
  - ◦ Systematize identification of health outcomes for mothers and offspring in the extended postnatal period and later in life –including women’s awareness of increased risk of non-communicable diseases such as chronic hypertension, cardiovascular and cerebrovascular disease.

The model can be adapted to different country contexts or tailored to country level. Here are the gaps to consider.

#### Critical issues to consider

The Primary Health Care Pre-eclampsia/ eclampsia (PHC PE/E) model (Fig. 1) displays the linkages and opportunities to prevent and treat PE/E in a simplified way; however, there are numerous interlinking factors, angles, and critical points to consider. The different elements of the model each play an important role and have different resource implications, when ensuring that women with PE/E are treated quickly and appropriately. Policies must be in place that include task sharing/shifting to PHC providers to prescribe antihypertensive drugs for managing moderate to severe hypertension (systolic BP ≥ 160 or diastolic ≥ BP-110) and administer the MgSO_4_ loading dose to women with severe PE/E. Standardized protocols need to be used all the time by everyone to ensure consistent management across the system [16, 22]. Leadership at facility, district, regional, national levels is critical to champion the implementation of the policy.

The medicines (MgSO_4_, calcium gluconate and antihypertensive drugs) recommended by WHO must be available where needed. Support to the public sector supply chain is required in order to have the right medicine, in the right place, at all times – and availability in the private sector or alternatives for when either system fails. Health care providers at PHC level must have the necessary equipment and logistics and the knowledge and skills to identify, provide initial treatment (loading dose of MgSO_4_ and antihypertensive) and refer [14, 24]. At referral facilities, providers should have the expertise and capacity for optimal emergency management of PE/E. Providers should benefit from refresher training, emergency drills/updates as needed, ongoing supportive supervision, and mentoring. However, it is important to recognize that task shifting or sharing to lower cadres only makes sense if those cadres already have basic clinical skills (for example taking BP, administering injections) [22, 23]. Evidence shows that MgSO_4_ should not be ‘pushed’ to the lowest level in communities (i.e. community health workers) but stay within a clinical setting [25]. We cannot forget that the women and communities must be involved, they must be knowledgeable and able to advocate for themselves and to ensure providers are listening to them. It is important that they recognize the danger signs, attend ANC in the first trimester and have at least eight contacts to be screened for signs of PE in order to minimize the impact of this disease on themselves and their newborns. Community platforms – such as women’s groups - can be leveraged to raise awareness about health topics, including PE/E [26, 27]. Referral pathways must be well defined to achieve the best outcomes. During training, cadres (from consultants to lower level providers) at all levels require updating in the referral process to ensure women receive prompt care. Close monitoring of women during labor and delivery is critical as is monitoring during the immediate postpartum period when 20–30% of hypertensive and eclamptic seizures occur and again before she leaves the facility, followed by comprehensive follow-up PNC. This PHC model complements the International Society for the Study of Hypertension in Pregnancy (ISSHP) recommendations for low resource settings [24] and resonates with new guidelines in South Africa [28].

This project has been pushing the agenda forward to provide better care for women on HDPs and PE/E and has an expanded view of what is needed [26, 27, 29, 30]. Ensuring services are available at lower levels providers opportunities to identify pre-eclampsia early, timely delivery and effective treatment for those with a severe form of the condition [14]. The results of the implementation research linked to this PHC model will be published as separate manuscripts. We are also considering reviewing the cost-effectiveness. If the mission of a public health system is to protect and improve pregnant women and their babies’ healthcare, it will be imperative for Ministries of Health to act comprehensively in addressing the issues of PE/E and MNH more broadly and specifically at PHC level [15, 18, 24, 30].

## Conclusion

The PHC model described here uses PE/E as an entry to improve the quality of ANC and by extension the pregnancy continuum. Bringing preventive and treatment services nearer to where pregnant women live makes sense. The global community have recognized this by re-endorsing PHC through the 2018 Astana commitment. Implementation research suggests that this PHC- PE/E model works and can improve the current and future health of women and infants.

## Data Availability

Not applicable.

## Abbreviations

ANC: antenatal care
BP: blood pressure
CMWs: community midwives
HDP: hypertensive disorders of pregnancy
MgSO_4_: magnesium sulphate
PE/E: preeclampsia and eclampsia
PHC: primary health care
PNC: postnatal care
PPH: postpartum hemorrhage
SDG: Sustainable Development Goal
